# 短链氯化石蜡对人体正常肝细胞的代谢干扰

**DOI:** 10.3724/SP.J.1123.2023.10037

**Published:** 2024-02-08

**Authors:** Yun LUO, Ningbo GENG, Shuangshuang CHEN, Lin CHENG, Haijun ZHANG, Jiping CHEN

**Affiliations:** 1.临沂大学医学院, 山东 临沂 276005; 1. College of Medicine, Linyi University, Linyi 276005, China; 2.中国科学院大连化学物理研究所, 中国科学院分离分析化学重点实验室, 辽宁 大连 116023; 2. CAS Key Laboratory of Separation Sciences for Analytical Chemistry, Dalian Institute of Chemical Physics, Chinese Academy of Sciences, Dalian 116023, China

**Keywords:** 液相色谱, 质谱, 短链氯化石蜡, 肝细胞, 代谢组学, 脂质代谢, 核苷酸代谢, liquid chromatography (LC), mass spectrometry (MS), short-chain chlorinated paraffins (SCCPs), hepatic cells, metabolomics, lipid metabolism, nucleotide metabolism

## Abstract

短链氯化石蜡(SCCPs)是一类新兴的持久性有机污染物,广泛存在于环境基质和人体样本中。SCCPs具有环境持久性以及远距离迁移能力,能够在生物体内积累并具有广泛的生物毒性效应,威胁着人类健康。本研究采用代谢组学方法评估了低剂量组(1 μg/L)、中剂量组(10 μg/L)以及高剂量组(100 μg/L)的SCCPs暴露对人体正常肝细胞L02的代谢干扰。主成分分析(PCA)与代谢扰乱水平指数(MELI)计算结果表明3个剂量组的SCCPs均能够引起L02细胞代谢活动的紊乱。在3个暴露组中,72个差异代谢物经二级质谱图信息定性或标准品验证。其中,1 μg/L SCCPs暴露组与10 μg/L SCCPs暴露组、100 μg/L SCCPs暴露组分别有33个、36个相同的差异代谢物。10 μg/L SCCPs暴露组与100 μg/L SCCPs暴露组有46个相同的差异代谢物。3个暴露组有33个相同的差异代谢物。在72个经二级质谱图信息定性或标准品验证的差异代谢物中,参与氨基酸代谢、核苷酸代谢和脂质代谢通路的差异代谢物分别有9、9以及45个。富集通路分析结果表明:SCCPs对L02细胞的代谢干扰主要表现在脂质代谢、脂肪酸β氧化以及核苷酸代谢通路上,且中、高剂量的SCCPs暴露引起更广泛的代谢通路的紊乱。SCCPs暴露干扰了甘油磷脂以及鞘脂类的代谢通路,其中,磷脂酰胆碱、磷脂酰乙醇胺以及鞘磷脂丰度的显著变化表明SCCPs暴露对细胞的生物膜有一定的损伤。同时,SCCPs暴露通过降低短链和中链酰基肉碱的含量抑制L02细胞中脂肪酸β氧化,提示细胞通过氧化脂肪酸供能减少。值得注意的是,与中剂量和低剂量SCCPs相比,高剂量的SCCPs暴露对脂肪酸β氧化的抑制作用更强。此外,SCCPs暴露诱导了核苷酸代谢通路的紊乱。次黄嘌呤水平显著升高提示SCCPs暴露可能诱导了L02细胞的缺氧、活性氧增多或者致癌等相关不良效应。

## Abstract (en)

Short-chain chlorinated paraffins (SCCPs) are an emerging class of persistent organic pollutants (POPs) that are widely detected in environmental matrices and human samples. Because of their environmental persistence, long-range transport potential, bioaccumulation potential, and biotoxicity, SCCPs pose a significant threat to human health. In this study, metabolomics technology was applied to reveal the metabolomic interference in human normal hepatic (L02) cells after exposure to low (1 μg/L), moderate (10 μg/L), and high (100 μg/L) doses of SCCPs. Principal component analysis (PCA) and metabolic effect level index (MELI) values showed that all three SCCP doses caused notable metabolic perturbations in L02 cells. A total of 72 metabolites that were annotated by MS/MS and matched with the experimental spectra in the Human Metabolome Database (HMDB) or validated by commercially available standards were selected as differential metabolites (DMs) across all groups. The low-dose exposure group shared 33 and 36 DMs with the moderate- and high-dose exposure groups, respectively. The moderate-dose exposure group shared 46 DMs with the high-dose exposure group. In addition, 33 DMs were shared among the three exposure groups. Among the 72 DMs, 9, 9, and 45 metabolites participated in the amino acid, nucleotide, and lipid metabolism pathways, respectively. The results of pathway enrichment analysis showed that the most relevant metabolic pathways affected by SCCPs were the lipid metabolism, fatty acid β-oxidation, and nucleotide metabolism pathways, and that compared with low-dose exposure, moderate- and high-dose SCCP exposures caused more notable perturbations of these metabolic pathways in L02 cells. Exposure to SCCPs perturbed glycerophospholipid and sphingolipid metabolism. Significant alterations in the levels of phosphatidylcholines, phosphatidylethanolamines, and sphingomyelins indicated SCCP-induced biomembrane damage. SCCPs inhibited fatty acid β-oxidation by decreasing the levels of short- and medium-chain acylcarnitines in L02 cells, indicating that the energy supplied by fatty acid oxidation was reduced in these cells. Furthermore, compared with low- and moderate-dose SCCPs, high-dose SCCPs produced a significantly stronger inhibition of fatty acid β-oxidation. In addition, SCCPs perturbed nucleotide metabolism. The higher hypoxanthine levels observed in L02 cells after SCCP exposures indicate that SCCPs may induce several adverse effects, including hypoxia, reactive oxygen species production, and mutagenesis in L02 cells.

氯化石蜡(chlorinated paraffins, CPs)是一类人工合成的氯代正构烷烃,其分子式为C_*n*_H_2*n*+2_Cl_*m*_,氯含量为30%~70%(质量分数)。根据碳链长度的不同,CPs可分为短链氯化石蜡(short-chain chlorinated paraffins, SCCPs, C_10_~C_13_)、中链氯化石蜡(medium-chain chlorinated paraffins, MCCPs, C_14_~C_17_)和长链氯化石蜡(long-chain chlorinated paraffins, LCCPs, C_18_~C_30_)^[1]^。因具有良好的热稳定性和化学稳定性,SCCPs被用作增塑剂和阻燃剂,并广泛添加在金属加工液、涂料、密封剂、黏合剂、皮革处理剂、塑料和橡胶中^[2,3]^。与MCCPs和LCCPs相比,SCCPs具有更低的相对分子质量、更高的蒸汽压和更高的水溶性,因此更容易释放到环境中^[4]^。由于SCCPs具有生物持久性以及远距离迁移能力、能够生物积累并带来一定的生物毒性,2017年斯德哥尔摩公约将其列入《关于持久性有机污染物的斯德哥尔摩公约》附件A的受控清单中^[5]^。此外,1990年国际癌症研究机构将平均碳链长度为12和平均氯化程度为60%的SCCPs归类为2B类潜在致癌物^[6]^。由于大规模生产和广泛的工业应用,SCCPs在各种环境介质、生物样本以及人体血液、母乳和脐带血中被频繁检出^[7⇓⇓⇓⇓-12]^。人体母乳和血液中SCCPs的污染水平是评估SCCPs健康风险的重要指标。据研究报道,在中国四川省收集的母乳中SCCPs含量为29.2~270.7 ng/mL^[11]^,在中国湖北省收集到的孕妇外周血中SCCPs含量为15.9~584 ng/mL^[11]^。因此,SCCPs对野生动植物和人类构成了潜在危害,对其进行生态风险和健康风险评估迫在眉睫。

据报道,SCCPs对水生生物、两栖动物、鸟类、哺乳动物、体外细胞以及土壤生物均产生多种毒性效应,如神经毒性、内分泌干扰效应、免疫调节效应、肝毒性、肾毒性、发育毒性、致死效应以及致癌效应^[13,14]^。研究者也广泛研究了SCCPs的毒性作用机制,他们发现SCCPs的毒性作用机制主要集中在氧化损伤、生物膜损伤、能量代谢抑制以及过氧化物酶体增殖物激活受体α(PPARα)激活、雌激素受体(ERα)激活、组成型雄烷受体(CAR)激活和β_2_肾上腺素能受体(β_2_-AR)等毒性作用通路的激活上^[1,15]^。高剂量SCCPs的长期暴露可能诱发多器官损伤。其中,肝脏是SCCPs最主要的毒性靶点。据文献[16]报道,暴露于SCCPs(1000 mg/kg)16天后,SD大鼠的肝脏明显增大,肝细胞高度脂肪变性,肝窦变窄,肝组织出现点状坏死,炎性细胞发生浸润,纤维组织出现增生。大鼠经SCCPs暴露后,诱发了肝脏过氧化物酶体增殖以及细胞质中脂肪滴数量增加^[17]^。目前,研究者对SCCPs肝毒性的研究集中在动物身上。SCCPs的致癌机制难以实现从啮齿类动物到人体的外推。如SCCPs对大鼠肝脏PPARα受体的激活作用和致肝癌有一定的关系。然而,PPARα受体在啮齿动物中高度表达,而在人体内是低表达的。因而,SCCPs通过PPARα受体的激活而产生致癌效应的机制在人体中可能并不适用^[18]^。因此,SCCPs对人体的毒性效应和机制研究有待进一步开展。

在过去的十几年中,组学技术(如转录组学、蛋白质组学和代谢组学)的重大进步使得研究者对各种生物过程的生物分子的高通量监测成为可能。组学技术在揭示外界因素作用于生物体的机制方面显现出了极其重要的作用,是毒性机制机理研究可以依赖的技术手段^[19,20]^。为评估SCCPs暴露对人体的潜在健康风险,本研究以人体正常肝细胞L02为模式细胞,以代谢组学技术为研究手段,从分子水平上探究了SCCPs肝毒性的代谢干扰机制。

## 1 实验部分

### 1.1 仪器、试剂与材料

Q-TOF 6540超高效液相色谱-四极杆飞行时间质谱仪(美国Agilent公司);Q-Trap 5500超高效液相色谱(美国Waters公司)-四极杆线性离子阱质谱仪(美国AB SCIEX公司);氧化碳培养箱(美国Thermo Fisher公司);台式高速低温离心机(Biofuge^©^ Stratos,德国Heraeus公司);倒置显微镜(南京江南光电股份有限公司);真空冷冻干燥仪(美国LABCONCO公司);涡旋振荡仪(G-560E,美国Scientific Industries公司)。

L02细胞购自中国科学院细胞库。SCCPs(C_10_~C_13_, 56.5%Cl)标样为C_10_-、C_11_-、C_12_-、C_13_-CP的1∶1∶1∶1(物质的量比)的混合标样,平均氯含量(以质量分数计)为56.5%,按照Tomyet等^[21]^报道的正烷烃氯化的方法在实验室合成。细胞培养所需试剂均购自美国Gibco公司,包括:细胞培养基(RPMI 1640)、胎牛血清、青霉素-链霉素、胰蛋白酶以及PBS缓冲溶液。甲醇和乙腈均为质谱纯级别,均购自Fisher Scientific公司(美国)。色谱级甲酸与色谱级碳酸氢铵购自百灵威公司(中国);细胞培养级别二甲基亚砜(DMSO)和D5-L-苯丙氨酸与辛酰基(8,8,8-D3)-L-肉碱购自Sigma-Aldrich公司(美国);十一烷酸(FFA C11∶0)与十九烷酸(FFA C19∶0)购自阿拉丁公司(中国); 1-十二烷基溶血磷脂酰胆碱(LysoPC 12∶0)与磷脂酰乙醇胺(PE 17∶0/17∶0)购自AVANTI公司(美国)。实验用水均为超纯水。

### 1.2 细胞暴露实验

将L02细胞置于培养箱中培养,将细胞培养箱的温度与二氧化碳体积分数设置为37 ℃与5%; L02细胞培养液为含有10%胎牛血清和1%青霉素-链霉素的RPMI 1640培养基。待细胞进入对数生长期时,将L02细胞接种在六孔板中,细胞接种密度为5×10^5^细胞/孔,并设置一个对照组与3个暴露组,每组设置6个平行。待细胞铺满六孔板面积的80%~90%,将细胞培养基弃去,向对照组加入含有0.05%(v/v) DMSO的培养液;将SCCPs(C_10_~C_13_, 56.5%Cl)通过DMSO引入培养基中,向3个暴露组分别加入含1 μg/L(低剂量)、10 μg/L(中剂量)、100 μg/L(高剂量)SCCPs的培养基,使DMSO在培养液中的最终体积分数为0.05%。3个暴露剂量均低于SCCPs在人体血液中的最高含量(584 ng/mL)^[11]^。

### 1.3 细胞代谢物提取

暴露24 h后,弃去六孔板中剩余细胞培养液,每孔加入2 mL超纯水快速洗涤细胞,洗涤3次后,加入液氮淬灭,使细胞的代谢活动终止,并放置于-80 ℃冰箱中保存,待进一步提取。

向六孔板的每孔中加入1 mL超纯水,冰水浴中超声3 min,使细胞破碎并从培养皿壁上脱落。随后将细胞碎片悬液转移到1.5 mL离心管中。将细胞破碎悬液冷冻干燥,然后加入0.5 mL 80%甲醇水溶液(其中含有6个内标,分别为0.5 μg/L D5-L-苯丙氨酸、0.5 μg/L辛酰基(8,8,8-D3)-L-肉碱、0.5 μg/L FFA C11∶0、0.5 μg/L FFA C19∶0、0.5 μg/L LysoPC 12∶0与1 μg/L PE 17∶0),室温涡旋20 min,离心(14000 g, 4 ℃)20 min。最后,取上清液过有机相滤膜并转移到进样小瓶中保存,待进行小分子代谢物分析。

### 1.4 代谢物检测

采用拟靶向代谢组学方法对细胞内小分子代谢物进行检测分析^[22]^。首先用Q-TOF 6540超高效液相色谱-四极杆飞行时间质谱对细胞样品进行非靶向分析,分为正、负离子两种扫描模式。通过高分辨Q-TOF-MS的AutoMS/MS功能对小分子代谢物实行全扫和二级质谱扫描,获得离子对信息。然后,采用Q-Trap 5500超高效液相色谱-四极杆线性离子阱质谱对获得的离子对进行靶向(Schedule MRM)分析,色谱分离条件与非靶向分析完全一致。

色谱条件 正离子模式:色谱柱为ACQUITY UPLC BEH C8(100 mm×2.1 mm, 1.7 μm, Waters, USA);流动相由0.1%甲酸水溶液(A)和乙腈(B)组成;梯度洗脱程序:0~1.0 min, 5%B; 1.0~23.0 min, 5%B~100%B; 23.0~27.0 min, 100%B; 27.0~27.1 min, 100%B~5%B; 27.1~30.0 min, 5%B。负离子模式:色谱柱为ACQUITY UPLC HSS T3 (100 mm×2.1 mm, 1.8 μm, Waters, USA);流动相由5 mmol/L NH_4_HCO_3_水溶液(A)与含5 mmol/L NH_4_HCO_3_的甲醇溶液(B)组成;梯度洗脱程序:0~1.0 min, 2%B; 1.0~3.0 min, 2%B~42%B; 3.0~12.0 min, 42%B~100%B; 12.0~16.0 min, 100%B; 16.0~17.0 min, 100%B~2%B; 17.0~20.0 min, 2%B。正、负离子模式下柱温均为50 ℃,流速均为0.35 mL/min。

Q-TOF 6540质谱参数 干燥气温度:350 ℃;干燥气流速:9 L/min;雾化器(N_2_气)压力:310.275 kPa (45 psi);毛细管电压:3500 V;碎裂电压:175 V;锥孔电压:65 V;MS采集范围: *m/z* 50~1000;MS/MS采集范围:*m/z* 30~1000;MS采集速率:4 spectra/s;MS/MS毛细管电压:2 spectra/s;碰撞能采用公式CE=5×*m/z*(母离子)/100+3计算;每个循环(0.25 s)允许分析的最大母离子个数设置为5,绝对阈值为5000 Count,相对阈值为1%。

Q-Trap质谱条件的正、负离子模式参数 电喷雾离子源(ESI),温度为550 ℃,喷雾电压分别为5500 V(正离子模式)与4500 V(负离子模式),气帘气压力为0.241 MPa,雾化气(ion source gas 1, GS1)压力为0.276 MPa,辅助气(ion source gas 2, GS2)压力为0.276 MPa。

### 1.5 数据处理及统计分析

使用MultiQuant软件(AB SCIEX)进行数据批量处理,导出经内标校正过的峰面积数据(某个样品中的原始峰面积除以该样品中内标峰面积再乘以所有样品内标峰面积的平均值),并进行归一化处理,用于后续的统计分析。MetaboAnalyst 5.0(http://www.metaboanalyst.ca/)网站用于单因素方差分析(ANOVA)、*t*-test以及主成分分析(PCA); RStudio(version 4.1.2)用于热图绘制;InCroMAP软件(version 1.7.0)用于通路富集分析。

此外,本研究还根据文献[23],进行了代谢扰乱水平指数(MELI)计算,来反映污染物暴露对代谢的整体干扰。首先计算单个样本中每个代谢物的代谢变化,然后将单个样本中所有代谢物的代谢变化进行求和,再除以样本中代谢物的总个数,从而得到该样本的MELI值。

### 1.6 质量控制与质量保证

在所有暴露组与对照组的样品中移取等量样品混合作为质控样品(QC)。将QC插入分析序列,每分析6个样品后分析一个质控样品。正、负离子模式下,各插入7个QC样品。计算QC样品中每个代谢物浓度的相对标准偏差(RSD),结果表明,在正、负离子模式下,一共检测到565个代谢物。其中,在正离子模式下检测到355个代谢物,93.2%的代谢物RSD<30%; RSD<10%、10%<RSD<20%与20%<RSD<30%的代谢物数量占总数的百分比分别为34.9%、44.5%与13.8%,只有6.8%的代谢物RSD>30%。在负离子模式下检测到210个代谢物,94.3%的代谢物RSD<30%。其中,RSD<10%、10%<RSD<20%与20%<RSD<30%的代谢物数量占总数的百分比分别为9.0%、54.8%与10.5%,只有5.7%的代谢物RSD>30%。该结果表明:代谢物靶标分析序列具有满意的稳定性和重复性。

## 2 结果与讨论

### 2.1 代谢轮廓分析

采用拟靶向代谢组学分析方法研究SCCPs暴露对L02细胞中小分子代谢物的扰乱,共检测到565个代谢物。为评估SCCPs引起的L02细胞中的代谢紊乱,对检测到的所有代谢物进行PCA分析(图1a),建立了包含第一主成分和第二主成分的二维空间模型。在PCA因子得分图中(图1a), 3个暴露剂量组在第一主成分上均能够与对照组明显分开。对照组分布在第二象限,低剂量组(1 μg/L)和中剂量组(10 μg/L)主要分布在第一象限,高剂量组(100 μg/L)主要分布在第三象限。表明3个剂量组的SCCPs均能够引起L02细胞代谢活动的紊乱;同时研究还发现,1 μg/L的SCCPs暴露组与10 μg/L的SCCPs暴露组部分重叠,表明1 μg/L和10 μg/L的SCCPs对L02细胞的代谢扰乱存在相似之处。为比较3个剂量组的SCCPs暴露对L02细胞总代谢的干扰强度,我们还计算了MELI。如图1b所示,3个暴露组的MELI值均显著(*P*<0.05)高于对照组。结果表明,3个剂量的SCCPs暴露均能引起L02细胞代谢紊乱。

**图1:**
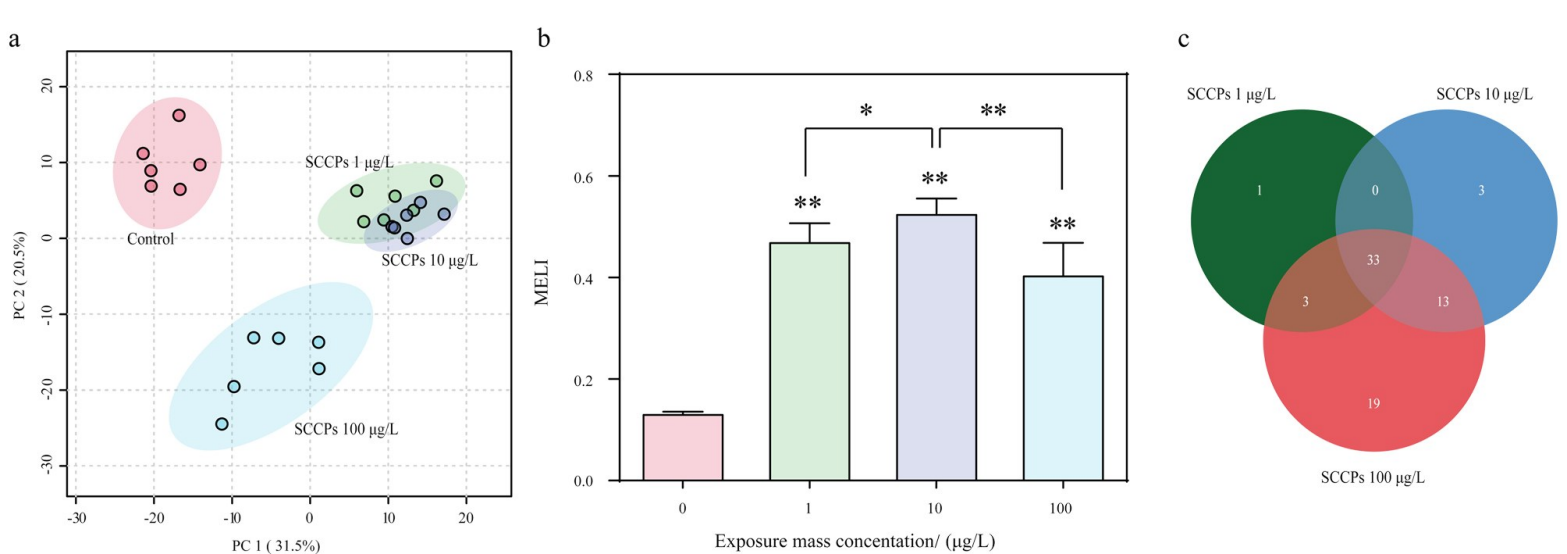
SCCPs暴露L02细胞24 h后引起的代谢扰动

通过MetaboAnalyst 5.0对所有暴露组与对照组的565个代谢物进行了ANOVA分析来筛选差异代谢物,筛选标准为False discovery rate (FDR)<0.05且Variable importance in the projection (VIP)值>1,共筛选出220个差异代谢物。其中,72个差异代谢物经化合物二级质谱图信息定性或标准品验证。韦恩图(图1c)显示,1 μg/L SCCPs暴露组与10 μg/L SCCPs暴露组和100 μg/L SCCPs暴露组分别有33个和36个相同的差异代谢物。10 μg/L SCCPs暴露组与100 μg/L SCCPs暴露组有46个相同的差异代谢物。3个暴露组有33个相同的差异代谢物。

为了进一步分析SCCPs暴露引起的细胞内代谢变化,基于各自的代谢通路类别,对所有定性的差异代谢物进行热图分析(图2)。在72个经二级质谱图信息定性或标准品验证的差异代谢物中,参与氨基酸代谢、核苷酸代谢和脂质代谢通路的差异代谢物分别为9、9以及45个。如图2所示,3个剂量的SCCPs暴露均引起参与氨基酸、核苷酸以及脂质代谢通路的代谢物的紊乱。

**图2:**
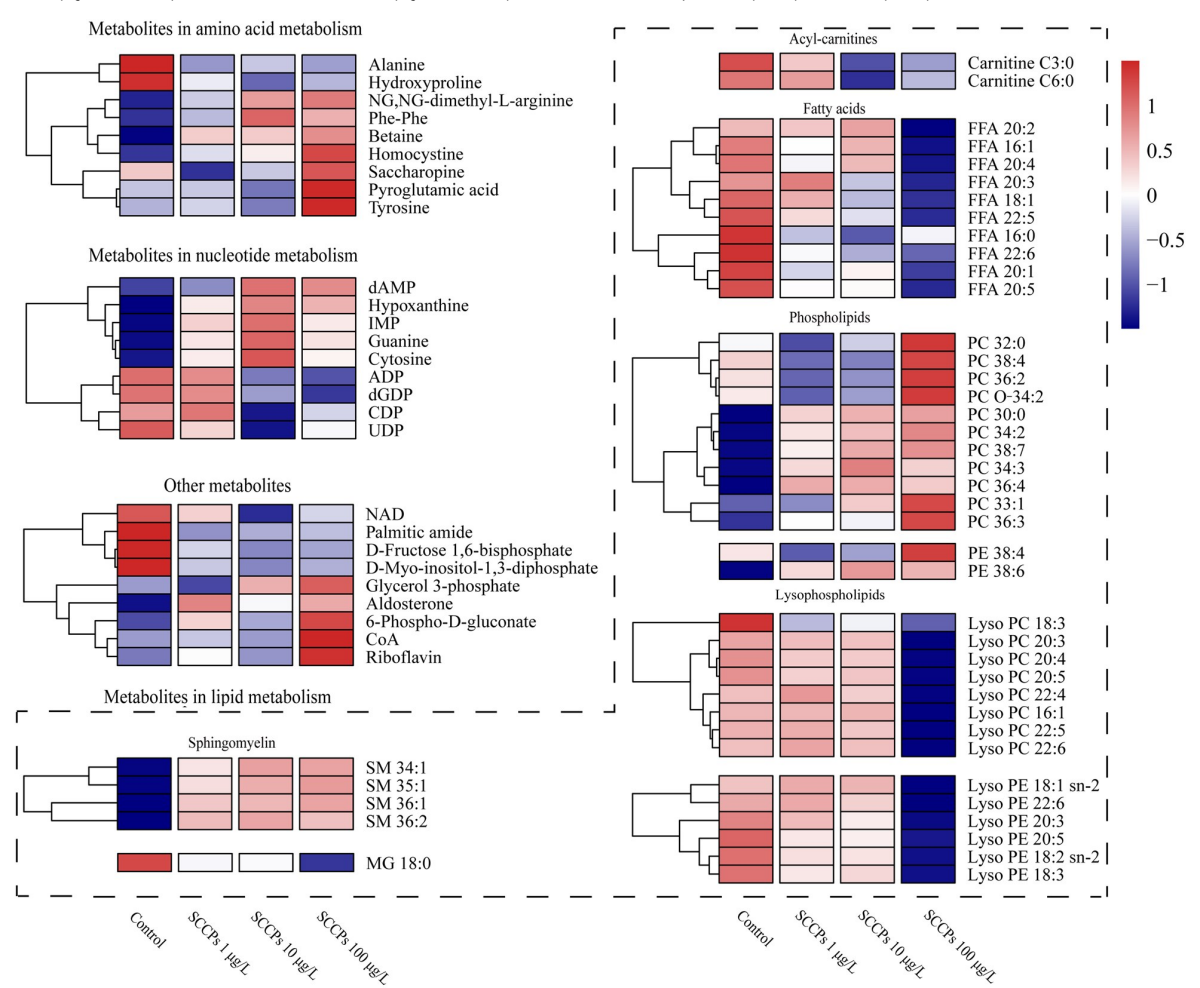
对照组和SCCPs暴露组中72个差异代谢物的热图

### 2.2 代谢通路分析

为了准确描述SCCPs暴露后L02细胞中代谢通路的改变,基于差异代谢物的倍率变化(FC)数据集对所有暴露组进行通路富集分析。保留与代谢相关且*P*<0.01的代谢通路作为显著影响的通路。-log_10_*Q*的数值代表SCCPs对代谢通路的干扰程度。如图3所示,低、中、高剂量的SCCPs暴露干扰了相似的代谢通路,包括甘油磷脂代谢、亚油酸代谢、α-亚油酸代谢、花生四烯酸代谢、不饱和脂肪酸合成通路、鞘脂类代谢以及嘌呤代谢通路。此外,随着暴露剂量的增加,SCCPs扰乱的代谢通路也同步增加,中、高剂量的SCCPs暴露还干扰氧化磷酸化(OXPHOS)以及脂肪酸合成通路。中剂量的SCCPs暴露单独干扰嘧啶代谢通路,高剂量的SCCPs暴露单独干扰苯丙氨酸、酪氨酸和色氨酸的生物合成通路。总之,中、高剂量的SCCPs暴露引起了更广泛的代谢通路紊乱。

**图3:**
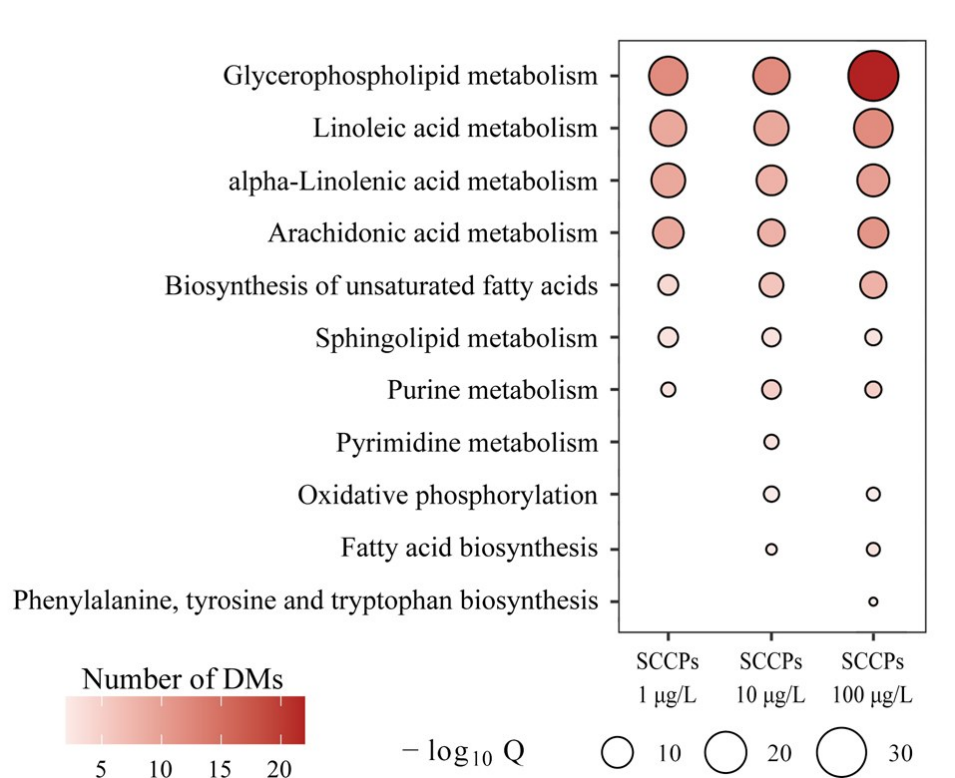
通路富集分析显示的SCCPs暴露后显著干扰的代谢通路

#### 2.2.1 脂质代谢通路干扰

脂质代谢是3个剂量SCCPs暴露组共同干扰的代谢通路,如图3所示,低、中、高剂量的SCCPs暴露均干扰甘油磷脂代谢以及鞘脂类代谢通路。SCCPs暴露后,脂类丰度发生了明显变化,包括磷脂酰胆(PCs)、磷脂酰乙醇胺(PEs)、溶血磷脂酰乙醇胺(lyso PEs)、溶血磷脂酰胆碱(lyso PCs)以及鞘磷脂(SMs)(图4)。PCs、PEs和SMs是细胞膜脂质双分子层的主要结构成分^[24]^,其丰度的显著变化表明SCCPs暴露对细胞膜有一定的损伤作用。已有研究表明:SCCPs可通过磷脂水平紊乱、结构亲脂性以及脂质过氧化破坏生物膜^[15]^。磷脂是细胞膜的重要组成部分,SCCPs暴露后磷脂水平的紊乱将会对细胞膜的稳定性和膜结合蛋白质的能力产生负面影响^[25]^。另外,磷脂的异常代谢还可以影响许多生物学过程,包括炎症效应^[26]^。

**图4:**
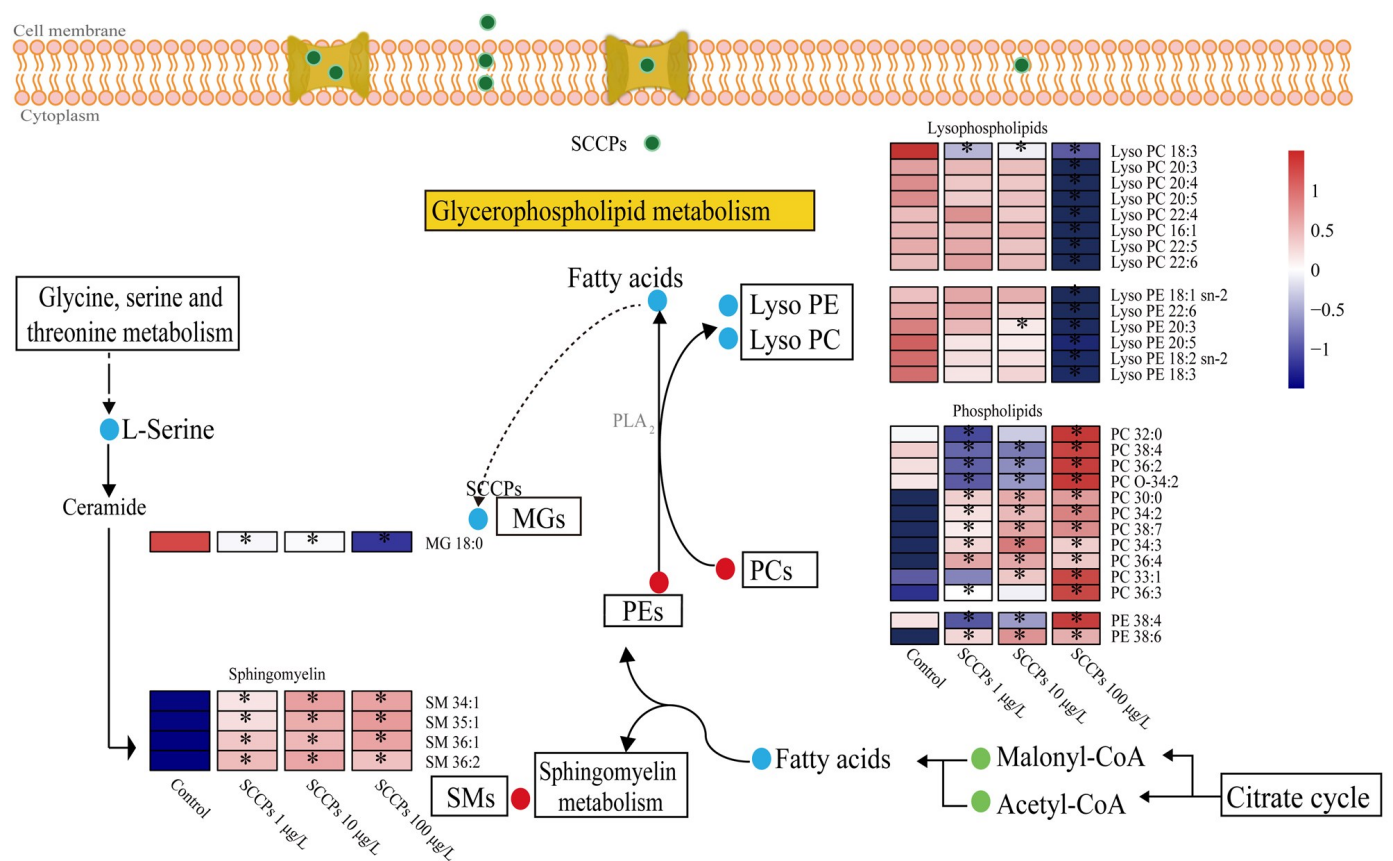
SCCPs诱导的脂质代谢通路的紊乱

低、中、高剂量的SCCPs均干扰了L02细胞中脂肪酸代谢,尤其是脂肪酸β氧化(图4)。如图5所示,短链(C3)、中链(C6)酰基肉碱的含量在中、高剂量的SCCPs暴露组中显著降低。同时,脂肪酸水平在3个剂量的SCCPs暴露组中均显著下降。

**图5:**
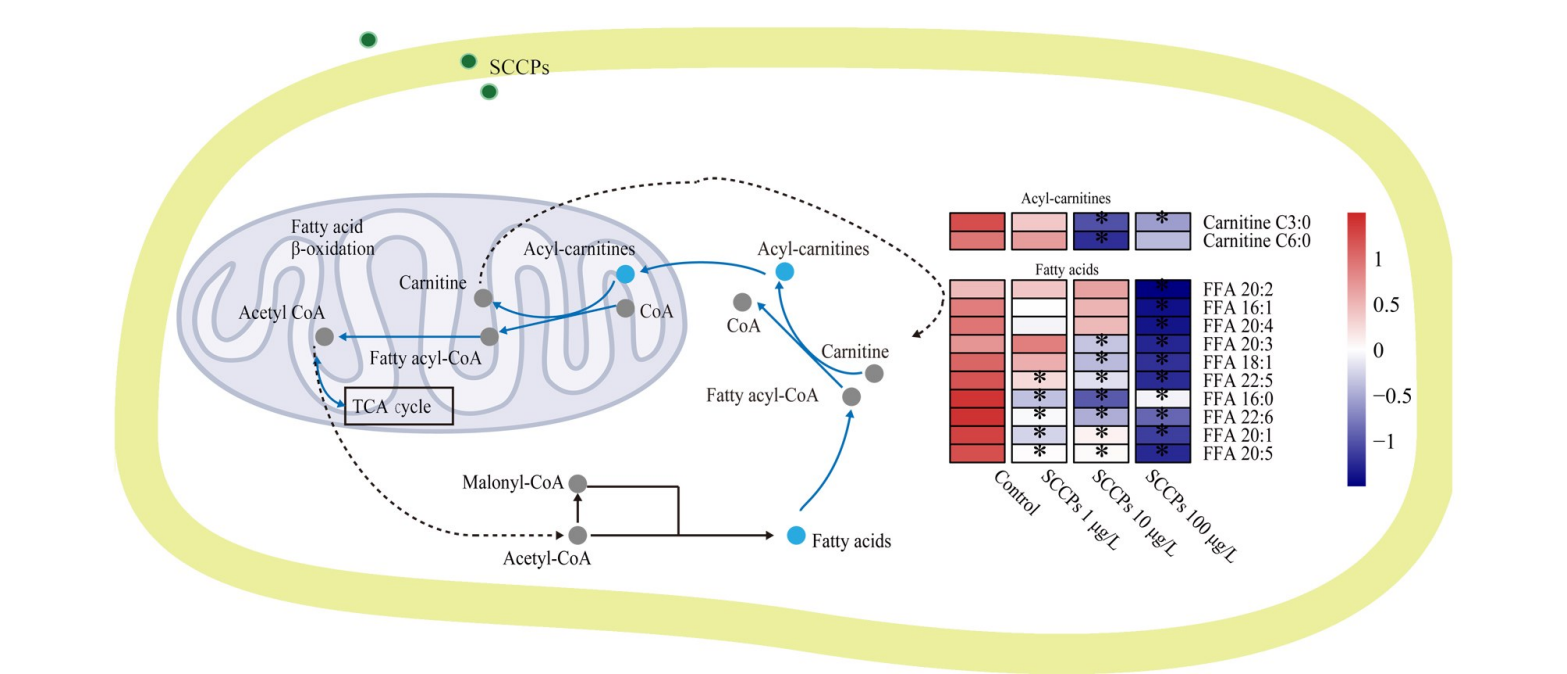
SCCPs诱导的脂肪酸代谢通路的紊乱

在脂肪酸β氧化过程中,脂肪酸首先被活化成脂酰CoA,而活化的脂酰CoA需进入线粒体才能被氧化。游离肉碱作为载体将脂肪酸运输到线粒体,并将其转化为相应的酰基肉碱,进而才能实现脂肪酸β氧化并产生能量^[27]^。因此,酰基肉碱的减少提示脂肪酸β氧化过程被抑制。在本研究中,SCCPs暴露降低了短链和中链酰基肉碱的含量,表明SCCPs暴露抑制了L02细胞中脂肪酸β氧化。值得注意的是,高剂量SCCPs对脂肪酸β氧化抑制作用更加明显。相关研究表明SCCPs对脂肪酸β-氧化通路的干扰可能与PPARα受体有关。SCCPs可通过激活大鼠肝脏PPARα受体,调控PPARα下游编码与脂肪酸代谢有关的蛋白质的靶基因的表达水平,进而影响线粒体脂肪酸β-氧化^[28]^。然而,PPARα激活效应存在物种差异,人PPARα的激活效应与啮齿类动物比相对较弱^[18,29]^。需要通过实验进一步探讨本研究中脂肪酸β氧化抑制作用与人PPARα的激活效应之间的关系。

#### 2.2.2 核苷酸代谢通路干扰

如图6所示,SCCPs暴露诱导了核苷酸代谢通路的紊乱。SCCPs暴露降低了L02细胞中二磷酸核酸(胞嘧啶核苷二磷酸(CDP)、尿苷二磷酸(UDP)、腺嘌呤核苷二磷酸(ADP)、脱氧鸟苷5'-二磷酸(dGDP))的含量。相反,SCCPs暴露诱导了单磷酸核苷(单磷酸肌苷(IMP)与脱氧腺苷单磷酸(dAMP))、鸟嘌呤(guanine)、胞嘧啶(cytosine)以及次黄嘌呤(hypoxanthine)的累积。核苷酸代谢失调会导致基因组不稳定和进一步的致癌作用^[30]^。据报道,次黄嘌呤水平的升高在许多器官系统中表现出有害的影响^[31,32]^。作为嘌呤降解通路中的反应中间体,次黄嘌呤经黄嘌呤氧化酶分解代谢成黄嘌呤和尿酸。在缺氧状态下,腺嘌呤核苷酸降解为次黄嘌呤的速度加快,黄嘌呤氧化酶转化次黄嘌呤为黄嘌呤和尿酸的速度减慢。因此,次黄嘌呤升高已被证明是几种疾病状态下缺氧的标志,比如围产期窒息、急性呼吸窘迫综合征、缺血性脑水肿^[33,34]^。另外,黄嘌呤氧化酶氧化次黄嘌呤也会产生活性氧(ROS)并引发细胞毒性。比如,次黄嘌呤已被证实通过ROS的产生上调胆固醇水平诱导动脉粥样硬化^[35]^。次黄嘌呤还可以在慢性炎症过程中通过腺嘌呤脱氨产生,能够启动炎症驱动效应并产生致癌作用^[36]^。在本研究中,低、中与高剂量的SCCPs暴露均导致了次黄嘌呤的累积,这与文献报道SCCPs对细胞产生氧化损伤的结果一致^[15]^。

**图6:**
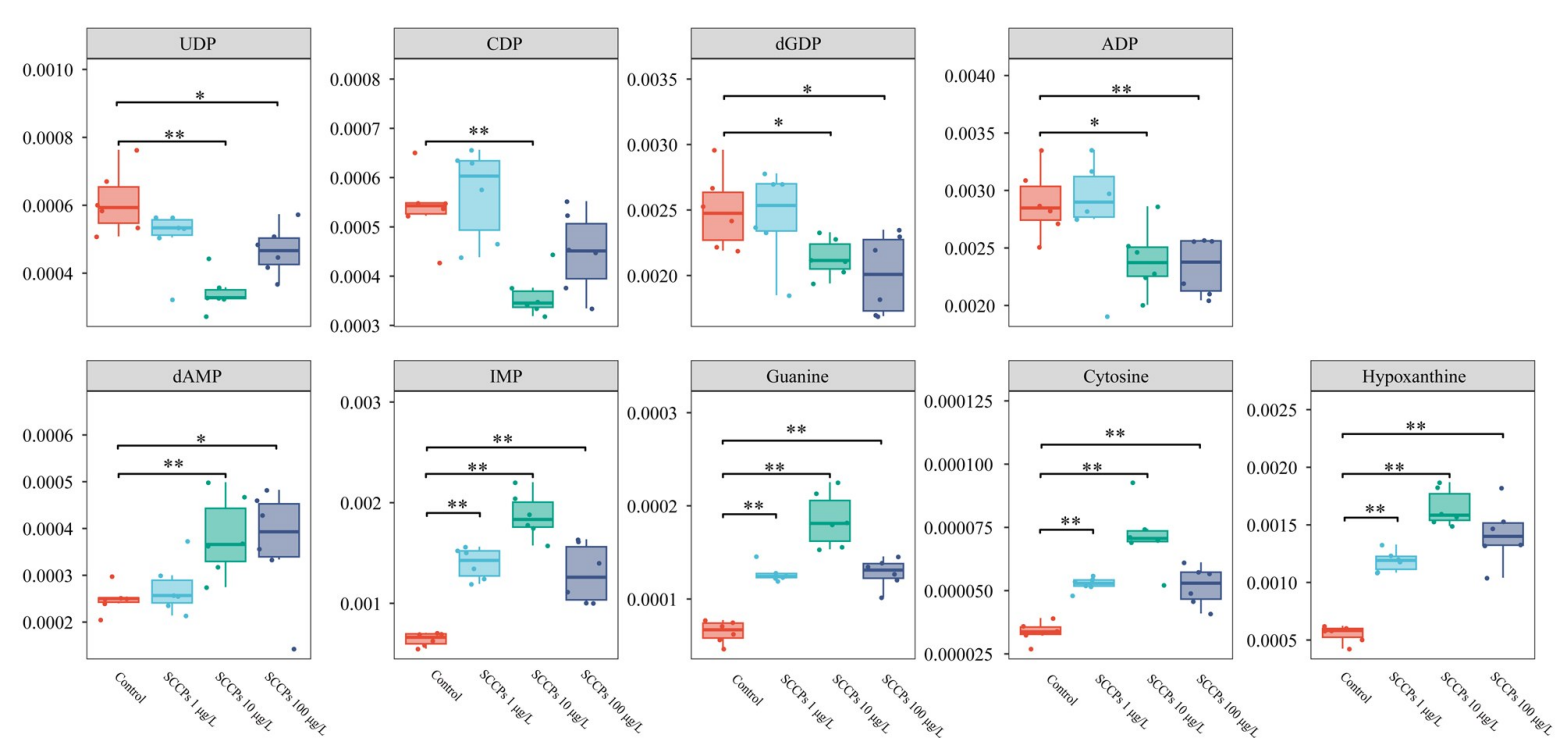
SCCPs暴露后核苷酸代谢通路相关代谢物的相对峰面积

## 3 结论

本研究以代谢组学为手段探讨了SCCPs暴露对人正常肝细胞L02的代谢干扰。研究表明:SCCPs对L02细胞的代谢干扰主要表现在脂质代谢、脂肪酸β氧化以及核苷酸代谢通路上。SCCPs暴露可能对细胞膜产生明显的损伤作用,抑制L02细胞中脂肪酸β氧化以及诱导L02细胞氧化损伤等相关不良效应。另外,在本研究中,与中、低剂量SCCPs暴露相比,高剂量SCCPs暴露引起更广泛的代谢通路的紊乱。同时,高剂量SCCPs暴露对脂质代谢、脂肪酸β氧化以及核苷酸代谢通路的干扰作用更强。然而,本研究仅仅在一个组学层面上来探索SCCPs对人体肝细胞的毒性机制显然是不够的,需要结合其他组学手段以及分子学技术手段深入探究其毒性作用机制,找到SCCPs对人体肝细胞的毒性作用靶点。
